# The Y-Chromosome Tree Bursts into Leaf: 13,000 High-Confidence SNPs Covering the Majority of Known Clades

**DOI:** 10.1093/molbev/msu327

**Published:** 2014-12-02

**Authors:** Pille Hallast, Chiara Batini, Daniel Zadik, Pierpaolo Maisano Delser, Jon H. Wetton, Eduardo Arroyo-Pardo, Gianpiero L. Cavalleri, Peter de Knijff, Giovanni Destro Bisol, Berit Myhre Dupuy, Heidi A. Eriksen, Lynn B. Jorde, Turi E. King, Maarten H. Larmuseau, Adolfo López de Munain, Ana M. López-Parra, Aphrodite Loutradis, Jelena Milasin, Andrea Novelletto, Horolma Pamjav, Antti Sajantila, Werner Schempp, Matt Sears, Aslıhan Tolun, Chris Tyler-Smith, Anneleen Van Geystelen, Scott Watkins, Bruce Winney, Mark A. Jobling

**Affiliations:** ^1^Department of Genetics, University of Leicester, Leicester, United Kingdom; ^2^Laboratory of Forensic and Population Genetics, Department of Toxicology and Health Legislation, Faculty of Medicine, Complutense University, Madrid, Spain; ^3^Molecular and Cellular Therapeutics, The Royal College of Surgeons in Ireland, Dublin, Ireland; ^4^Department of Human Genetics, Leiden University Medical Centre, Leiden, The Netherlands; ^5^Istituto Italiano di Antropologia, Rome, Italy; ^6^Department of Environmental Biology, Sapienza University of Rome, Rome, Italy; ^7^Division of Forensic Sciences, Norwegian Institute of Public Health, Oslo, Norway; ^8^Centre of Arctic Medicine, Thule Institute, University of Oulu, Oulu, Finland; ^9^Utsjoki Health Care Centre, Utsjoki, Finland; ^10^Department of Human Genetics, University of Utah Health Sciences Center, Salt Lake City, UT; ^11^Laboratory of Forensic Genetics and Molecular Archaeology, KU Leuven, Leuven, Belgium; ^12^Department of Imaging & Pathology, Biomedical Forensic Sciences, KU Leuven, Leuven, Belgium; ^13^Laboratory of Biodiversity and Evolutionary Genomics, Department of Biology, KU Leuven, Leuven, Belgium; ^14^Department of Neurosciences, University of the Basque Country, San Sebastián, Spain; ^15^National Center for Thalassemias, Athens, Greece; ^16^School of Dental Medicine, Institute of Human Genetics, University of Belgrade, Belgrade, Serbia; ^17^Department of Biology, Tor Vergata University, Rome, Italy; ^18^Network of Forensic Science Institutes, Institute of Forensic Medicine, Budapest, Hungary; ^19^Department of Forensic Medicine, Hjelt Institute, University of Helsinki, Helsinki, Finland; ^20^Department of Molecular and Medical Genetics, Institute of Applied Genetics, University of North Texas Health Science Center, Fort Worth, Texas; ^21^Institute of Human Genetics, University of Freiburg, Freiburg, Germany; ^22^Department of Molecular Biology and Genetics, Boğaziçi University, Istanbul, Turkey; ^23^Wellcome Trust Sanger Institute, Hinxton, Cambridge, United Kingdom; ^24^Laboratory of Socioecology and Social Evolution, Department of Biology, KU Leuven, Leuven, Belgium; ^25^Department of Oncology, University of Oxford, Oxford, United Kingdom

**Keywords:** Y-chromosome phylogeny, single nucleotide polymorphisms, targeted resequencing, Y-STRs, purifying selection

## Abstract

Many studies of human populations have used the male-specific region of the Y chromosome (MSY) as a marker, but MSY sequence variants have traditionally been subject to ascertainment bias. Also, dating of haplogroups has relied on Y-specific short tandem repeats (STRs), involving problems of mutation rate choice, and possible long-term mutation saturation. Next-generation sequencing can ascertain single nucleotide polymorphisms (SNPs) in an unbiased way, leading to phylogenies in which branch-lengths are proportional to time, and allowing the times-to-most-recent-common-ancestor (TMRCAs) of nodes to be estimated directly. Here we describe the sequencing of 3.7 Mb of MSY in each of 448 human males at a mean coverage of 51×, yielding 13,261 high-confidence SNPs, 65.9% of which are previously unreported. The resulting phylogeny covers the majority of the known clades, provides date estimates of nodes, and constitutes a robust evolutionary framework for analyzing the history of other classes of mutation. Different clades within the tree show subtle but significant differences in branch lengths to the root. We also apply a set of 23 Y-STRs to the same samples, allowing SNP- and STR-based diversity and TMRCA estimates to be systematically compared. Ongoing purifying selection is suggested by our analysis of the phylogenetic distribution of nonsynonymous variants in 15 MSY single-copy genes.

## Introduction

The male-specific region of the Y chromosome (MSY) has been widely exploited in studies of human evolution and population history (Jobling and Tyler-Smith 2003), but has suffered from ascertainment bias in the sequence variants studied. Also, although phylogenies constructed from such variants over the last two decades (Hammer 1995; Underhill et al. 2000; Y Chromosome Consortium 2002; Karafet et al. 2008) have been useful in defining haplogroups whose distributions can be investigated in different populations, nodes could not be dated directly from sequence variation. Consequently, dating has generally relied on use of another class of marker, Y-specific short tandem repeats (STRs). While being comparatively free from ascertainment bias because they are variable in all populations, these markers are affected by uncertainty over the appropriate choice of mutation rate (Zhivotovsky et al. 2004). There are also possible problems due to mutation saturation over long time-scales (Busby et al. 2012; Wei, Ayub, Xue, et al. 2013), and to differences among STRs in repeat motif and array length, and array inhomogeneity (Ballantyne et al. 2010).

Application of next-generation sequencing (NGS) to large segments of the MSY can provide unbiased ascertainment of single nucleotide polymorphisms (SNPs) and allows detailed phylogenies to be constructed in which branch-lengths are proportional to time, allowing direct assessment of the times-to-most-recent-common-ancestor (TMRCAs) of nodes. Recently, five NGS-based trees (1000 Genomes Project Consortium et al. 2010; Francalacci et al. 2013; Poznik et al. 2013; Wei, Ayub, Chen, et al. 2013; Scozzari et al. 2014) have been described (table 1), providing insights into events in human evolution and population relationships from a patrilineal perspective. However, these trees vary greatly in their sample sizes (from 36 to 1,208 Y chromosomes), their number of population samples (from 1 to 9), and their representation of known lineages. Sequencing methodologies have also been heterogeneous, with consequent variation in the amount of DNA sequenced (from 1.5 to ∼10 Mb) and mean coverage (from 2× to 50×). In low-coverage approaches, imputation methods have been employed to infer the allelic states of missing genotypes based on the phylogeny itself, and singletons (variants present only once in the data set) that define the lengths of terminal branches have been poorly ascertained, with consequent likely underestimation of branch lengths (Francalacci et al. 2013).

**Table 1.** NGS Studies of Human Y-Chromosome Diversity.

Study	Approach	Mb	Mean Read Depth	*n*	Sample Choice	SNPs Imputed?	SNPs Found	Overlap with This Study (%)
1000 Genomes Project Consortium et al. (2010)	WGS	Unclear	1.8×	77	4 HapMap populations	No (ML tree)	2,870	635/13,261^a^ (4.79)
Wei, Ayub, Chen, et al. (2013)	WGS	8.97	28.4×	36	Various (Complete Genomics data set, plus hg A male)	Yes	5,865 (+56 MNPs, 741 indels)	1,776/13,261 (13.4)
Poznik et al. (2013)	WGS	9.9	Median 3.1× at var sites	69	9 populations (7 from HGDP)	Yes	11,640	2,420/13,261 (18.25)
Francalacci et al. (2013)	WGS	8.97	2.16×	1,208	Sardinian population	Yes	11,763 (no singletons)	2,229/13,261 (16.8)
Scozzari et al. (2014)	SC	1.50	50×	68	Phylogenetic	Not stated	2,386	665/13,261 (5.01)
This study	SC	3.7	51×	448	19 pops. + phylogenetic	No	13,261	Novel: 8,742/ 13,261 (65.9)

WGS, whole-genome sequence; SC, sequence capture; ML, maximum likelihood; HGDP, Human Genome Diversity Panel.

^a^Based on available file containing 2,788 variants in 75 individuals.

No single study has so far combined high-coverage sequencing of multimegabase segments of the MSY in a wide range of samples that covers the majority of the known clades of the phylogeny. Here we accomplish this, combining a phylogeny of 334 Y chromosomes (Batini et al. submitted) based on 17 populations of Europe and the Middle East, which we use elsewhere as a tool to interrogate the demographic history of European populations, with an additional 114 MSY sequences that together ensure that major clades and deep-rooting nodes are represented.

The resulting phylogeny, based on a mean coverage of 51× in 3.7 Mb from each of 448 Y chromosomes, contains 13,261 high-confidence SNPs. It resolves polytomies, provides date estimates for deep nodes, and constitutes a robust evolutionary framework for analyzing the history of other classes of mutation affecting the MSY, including Y–Y and X–Y gene conversion events and structural variants. We also analyze variation at a set of 23 Y-STRs in all 448 samples, allowing a systematic comparison of SNP- and STR-based diversity and TMRCA estimates. Analysis of damaging nonsynonymous variants in 15 single-copy genes with our sequenced regions shows an underrepresentation of shared variants, implying that purifying selection is active on MSY.

## Results

Elsewhere (Batini C, Hallast P, Zadik D, Maisano Delser P, Benazzo A, Ghirotto S, Arroyo-Pardo E, Cavalleri GL, de Knijff P, Dupuy BM, Eriksen H, King TE, López de Munain A, López-Parra AM, Milasin J, Novelletto A, Pamjav H, Sajantila A, Tolun A, Winney B and Jobling MA, submitted.) we have described an NGS-based MSY phylogeny based on 5,996 SNPs ascertained in 334 human Y chromosomes comprising 17 population samples from Europe and the Near East, focused on illuminating the origins and histories of European patrilineages. Here, we supplemented those data with additional MSY sequences from random selections of 20 males from each of two HapMap populations, YRI (Yorubans from Ibadan, Nigeria) and CHB (Han Chinese from Beijing), plus 74 males from various populations, known from previous analyses to carry Y chromosomes belonging to specific haplogroups, in order to ensure that major clades and deep-rooting nodes were represented. Supplementary table S1, Supplementary Material online, lists all the samples analyzed and their populations of origin. We simultaneously generated orthologous MSY sequences from 22 great ape males using the same experimental approach, which we use here as an outgroup to the human sequences, and which will be described fully elsewhere.

**Fig. 1.**
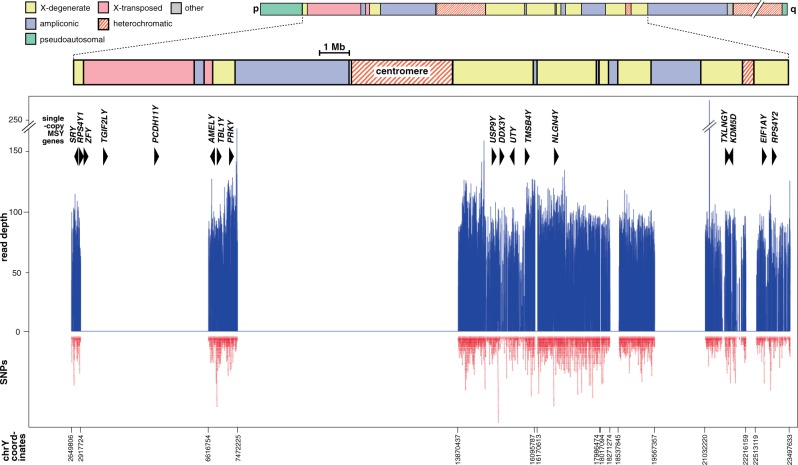
Distribution of sequenced regions on the MSY. At the top is shown a schematic representation of the Y chromosome and the analyzed subregion, with the distribution of the ampliconic, X-transposed, X-degenerate, and heterochromatic regions indicated (Skaletsky et al. 2003). The graph shows read depth in sequenced regions (blue) and density of discovered SNPs (red). Target coordinates for bait design (bottom) are according to GRCh37. Also shown are the locations of single-copy MSY genes (Skaletsky et al. 2003; Bellott et al. 2014), as triangles pointing in the direction of transcription. *TXLNGY* (Putative gamma-taxilin 2) replaces the former *CYorf15A* and *CYorf15B* (Skaletsky et al. 2003).

**Fig. 2.**
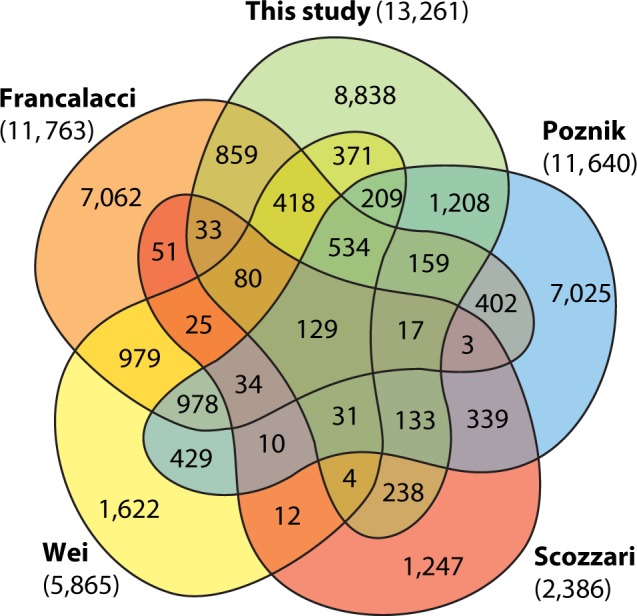
Venn diagram showing overlap of SNPs between NGS studies of the MSY. The total number of independent SNPs across all five studies (this study plus Francalacci et al. [2013], Poznik et al. [2013], Scozzari et al. [2014], and Wei, Ayub, Chen, et al. [2013]) is 33,479.

We used a sequence-capture design (see Materials and Methods) that allowed us to analyze 3.7 Mb of readily interpretable human MSY sequence, excluding the ampliconic and X-transposed regions (Skaletsky et al. 2003) of the chromosome (fig. 1; supplementary tables S2 and S3, Supplementary Material online), and gapped due to the repeat-masking required when designing sequence-capture probes. Mean coverage was 51×, and we called all SNPs (ignoring indels) with ≥6× coverage, validating in silico by comparison with published whole-genome sequence and genome-wide SNP chip data. The high coverage and high threshold for variant calling led, respectively, to low false-negative and false-positive rates (supplementary material, Supplementary Material online). We ascertained 13,261 SNPs, cross-referencing them with those identified in other published studies (fig. 2 and supplementary table S4, Supplementary Material online): Of our SNPs, 2,356 (17.8%) are in dbSNP build 138, 8,742 (65.9%) have not been previously reported, and over half are singletons, that is, unique in the data set (7,782; 58.7%).

### Features of the MSY Phylogeny and TMRCAs of Nodes

**Fig. 3.**
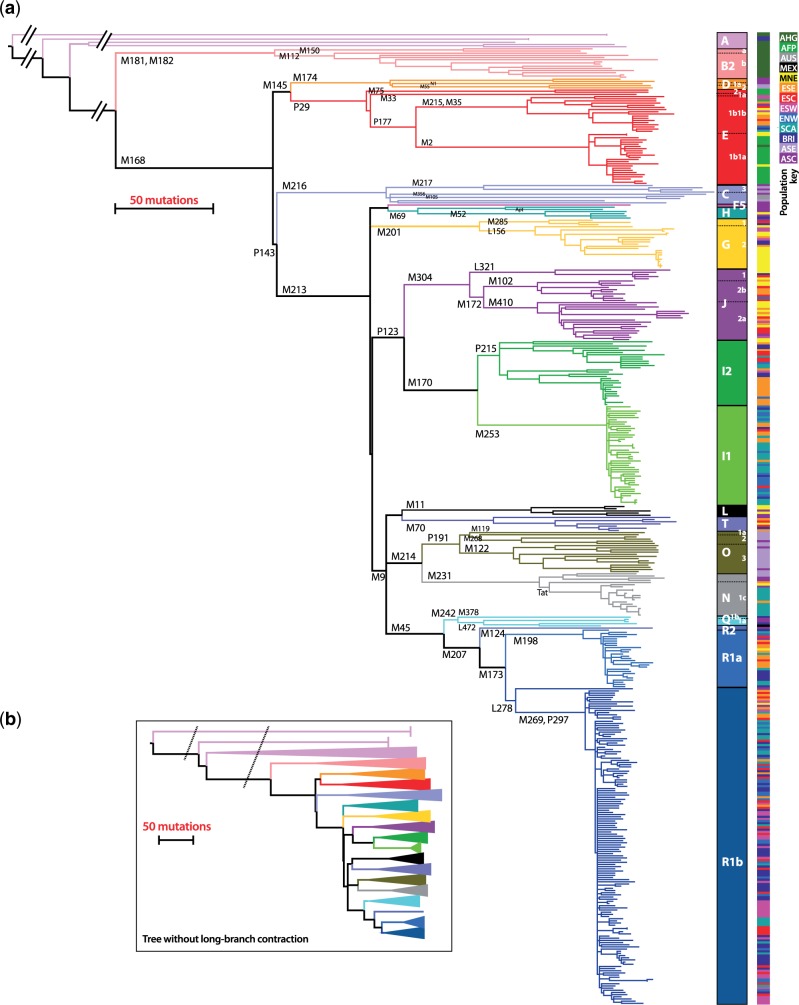
Maximum-parsimony tree of MSY SNP haplotypes. (*a*) Major haplogroups are indicated by colors, and selected haplogroup-defining mutations are indicated on branches. Deep-rooting branches have been contracted for display. The colored bar to the right indicates population group of origin: ASC: Asia, Central; ASE: Asia, East; BRI: British Isles; SCA: Scandinavia; ENW: Europe, North West; ESW: Europe, South West; ESC, Europe, South Central; ESE: Europe, South East; MNE: Middle and Near East; MEX: Mexico; AUS: Australia; AFP: Africa, food-producers; AHG: Africa, hunter-gatherers. Supplementary figure S1, Supplementary Material online, gives tips labeled with individual sample names. (*b*) Simplified tree showing the true lengths for deep-rooting branches. Diagonal dashed lines indicate the positions of branch contractions in part (*a*).

A maximum-parsimony tree (fig. 3 and supplementary fig. S1, Supplementary Material online) was built using the program PHYLIP (Felsenstein 2005), and rooted using great ape sequences generated using the same technical approach as the human sequences (see supplementary material, Supplementary Material online). We used the program AMY-tree (Van Geystelen et al. 2013a) to seek previously identified haplogroup-defining SNPs within our data set, and thus to name the major clades based on existing Y-haplogroup nomenclature (Karafet et al. 2008); for all 154 samples for which previously generated Y-SNP data were available, all such designations were consistent. We also applied a new version of AMY-tree (version 2.0) that considered only the targeted MSY regions to assess phylogenetic consistency, and thus to act as an additional quality-control for our data (supplementary material, Supplementary Material online). This approach revealed no calls ascribable to sequencing error in a total of 516,096 genotypes at 1,152 sites checked, confirming the high data quality. Our phylogeny contains all known top-level alphabetically labeled clades, with the exceptions of haplogroups A00 (Mendez et al. 2013), M and S. We used the rho statistic (Forster et al. 1996; Saillard et al. 2000) to estimate TMRCA for major nodes within the tree (table 2). Previously we (Batini et al. submitted) and others (Wei, Ayub, Xue, et al. 2013; Scozzari et al. 2014) have also employed the coalescent-based method implemented in BEAST (Drummond et al. 2005; Drummond and Rambaut 2007), but here our sampling violates the requirement of population sampling, so we focus on rho, noting that dates for NGS data estimated using both methods are strongly correlated (Scozzari et al. 2014; Batini et al. submitted). We use a pedigree-based MSY-specific mutation rate (Xue et al. 2009) and address the issue of mutation rate choice in the Discussion.

**Table 2.** TMRCA Estimates for Selected Clades within the Phylogeny.

Clade	*N*	TMRCA/ka	TMRCA Range Based on Mutation Rate CI/ka
Root	448	125.8	50.3–419.5
B-M182	14	45.6	18.2–152.0
B2a-M150	2	16.6	6.7–55.5
B2b-M112	12	38.1	15.2–127.1
CR-P143	378	47.9	19.2–159.8
C-M216	9	39.4	15.8–131.5
DR-M168	427	48.7	19.5–162.4
D-M174	5	34.3	13.7–114.4
DE-M145	49	48.1	19.2–160.3
E-P29	44	37.9	15.2–126.4
E1b1a-M2	24	6.9	2.7–22.9
E1b1b-M215	17	17.7	7.1–58.9
FR-M213	369	35.2	14.1–117.2
HF5	7	31.6	12.7–105.5
G-M201	23	23.1	9.2–77.0
G2a-L31	20	16.4	6.6–54.8
H-M69	6	27.4	11.0–91.5
I-M170	76	20.6	8.2–68.6
I1-M253	46	3.5	1.4–11.5
I2-P215	30	17.1	6.8–57.0
J-M304	33	23.3	9.3–77.7
IJ-P123	109	31.0	12.4–103.4
J2-M172	28	21.1	8.4–70.3
J2a-M410	18	15.2	6.1–50.8
J2b-M102	10	11.3	4.5–37.8
LT	12	32.6	13.1–108.8
L-M11	5	14.2	5.7–47.3
T-M70	7	21.0	8.4–70.1
M/LT/NO/QR-M9	230	32.6	13.0–108.6
NO-M214	39	30.0	12.0–99.9
N-M231	20	13.4	5.4–44.8
N1c1-M178	15	4.6	1.8–15.2
O-P191	19	25.6	10.2–85.3
P-M45	179	24.2	9.7–80.6
Q-M242	5	22.6	9.0–75.4
R-M207	174	19.3	7.7–64.4
R1a-M198	27	6.2	2.5–20.8
R1b-L278	146	14.3	5.7–47.7
R1b-M269	145	4.9	2.0–16.3

Here we comment on striking or novel features of the phylogeny and the point estimates of TMRCAs, beginning with the deeper-rooting nodes and then focusing on some specific clades:

- *Basal African clades:* The tree demonstrates the remarkable depth of MSY ancestry retained among hunter-gatherer groups in Africa within the rare haplogroups A and B. As has previously been observed (Poznik et al. 2013; Scozzari et al. 2014) the longest internal branches in the entire phylogeny (fig. 3*b*) are those among these clades, together with the branch that separates them from the remainder of the tree, superhaplogroup DR, corresponding to the expansion of Y chromosomes following the out-of-Africa migration (Underhill et al. 2000; Wei, Ayub, Chen, et al. 2013). Considering the point estimate of mutation rate, TMRCA for the entire tree is approximately 126 ka, and that for the DR node 49 ka, which correlates reasonably well with the date of the colonization of Eurasia.
- *Ancient population expansion:* Within clade DR of the tree lies a deep Paleolithic lineage radiation, giving rise to haplogroups G, HF5, IJ, LT, NO, and P, dating to between 23 and 33 ka (we note that a single variant identified elsewhere [Poznik et al. 2013] resolves the polytomy of haplogroups G and H, with G branching earlier).
- *Ancient subclades within hgs C and D:* The phylogeny also contains sequences within the largely Asian haplogroups C and D, which have not been sequenced elsewhere. In both cases, most branches are long, and the TMRCAs for the clades are similar, at 39 and 34 ka.
- *Bantu-speaking populations and expansions in hg E:* Within haplogroup E the most striking features are the shallow star-like genealogies within E1b1a, which predominate in the food-producing, Bantu-speaking YRI+LWK (Luhya in Webuye, Kenya), and present a stark contrast to the ancient hunter-gatherer lineages in A and B. Hg E1b1a (here given a TMRCA of 6.9 ka) has previously been associated with the expansion of Bantu languages, which spread widely from Central Africa approximately 3 ka together with farming and iron-working (Berniell-Lee et al. 2009).
- *A novel hg F sublineage associated with hg H*: One Nepalese sample had been previously assigned to hg F*, and here its branch (newly named F5) arises, with hg H, from a deep-rooting bifurcation with TMRCA 32 ka.
- *Hg H in Asia and English Romany:* The tree contains six sequences within haplogroup H, with a TMRCA of 27 ka. The clade is largely found in the Indian subcontinent, but is also typical of European Roma, who originated in a founder population from north/northwestern India approximately 1.5 ka (Mendizabal et al. 2011). One MSY sequence belongs to an English Romany male, previously assigned to haplogroup H1a (King TE, unpublished data), and here arising from a trifurcation with Turkish and Nepalese haplotypes.
- *Star-like expansion within hg I*: Haplogroups I and J divide at 31 ka, and each then divides into two at similar times of 21–23 ka. Within hg I1 is a striking star-like genealogy dating to 3.5 ka (Batini et al. submitted).
- *Rare deep-rooting hg Q lineages in NW Europe*: Hg Q has been most widely investigated in terms of the peopling of the Americas from NE Asia (Karafet et al. 1999). Here, as well as an example of the common native American Q-M3 lineage, we included examples of rare European hg Q chromosomes. One of the English chromosomes belongs to the deepest-rooting lineage within Q (Q-M378) and may reflect the Jewish diaspora (Hammer et al. 2009); the other is distantly related, shares a deep node with the Mexican Q-M3 chromosome, and has an STR-haplotype closely related to those of scarce Scandinavian hg Q chromosomes (King TE, Jobling MA, unpublished data).
- *Structure within the west Eurasian hg R*: The TMRCA of hg R is 19 ka, and within it both hgs R1a and R1b comprise young, star-like expansions discussed extensively elsewhere (Batini et al. submitted). The addition of Central Asian chromosomes here contributes a sequence to the deepest subclade of R1b-M269, whereas another, in a Bhutanese individual, forms an outgroup almost as old as the R1a/R1b split.

### SNP-Based Discrimination among Males and Comparison with Y-STRs

As with other NGS studies of MSY, our analysis reveals very high diversity compared with previously established phylogenies (Karafet et al. 2008). However, despite this high resolution, not all Y chromosomes in the sample can be distinguished. The 448 MSY sequences belong to 440 different SNP haplotypes, identical cases being found in eight pairs of individuals. One pair, within hg A1, belongs to a previously reported deep-rooting English pedigree (King et al. 2007), and the males are separated by just 13 generations. The remaining seven pairs are apparently unrelated, but each pair belongs to a single population—there are three identical Saami pairs (two within hg N1c1 and one in hg I1), two Palestinian pairs (in hgs E1b1b and G2a), one Bakola pair (hg A1b), and one Italian pair (hg R1b).

The traditional tools for discriminating between closely related Y chromosomes are Y-STRs. Identification of rapidly mutating STRs (RM-STRs) (Ballantyne et al. 2010) discriminates between brothers in 60% of cases (Ballantyne et al. 2012), so their application is expected to exceed the feasible resolution of NGS approaches. However, traditionally applied sets of STRs have lower average mutation rates than these. To investigate the relative discrimination power of SNP and STRs, we typed all 448 samples using PowerPlex Y23 (Promega), a 23-STR forensic multiplex kit that contains markers with varying mutation rates, including two RM-YSTRs (DYS570 and DYS576) (Purps et al. 2014). The resulting STR haplotypes also fail to discriminate among all samples, yielding six identical pairs (supplementary fig. S2*a*, Supplementary Material online), again each within-population (supplementary table S5, Supplementary Material online). Only two of these are also SNP-identical, the others being discriminated by 1–5 SNPs. Removal of the two RM-YSTRs leads to identical haplotypes in two additional pairs and a trio, separated by 1–3 SNPs, and still within-population. Removal of a further four STRs from the haplotype to reduce it to the 17 STRs contained in the Yfiler kit (Life Technologies) leads to identical haplotypes in an additional trio and three pairs of individuals, including two cross-population cases (Norway–Serbia and Serbia–Spain), and up to 31 SNPs separating members of a pair. This emphasizes the homoplasic nature of STR haplotypes (Larmuseau et al. 2014), and the importance of analyzing many STRs for forensic identification and genealogical purposes.

### Comparison of SNP- and STR-Based TMRCA Estimates

Y-STRs have also been widely employed for dating purposes, and here we used our Y-STR data to estimate TMRCAs (supplementary table S6, Supplementary Material online) for the lineages dated with SNPs, allowing us to compare the two marker types. We explored three variables:

- Two different dating methods—rho (Forster et al. 1996; Saillard et al. 2000) and average-squared distance (ASD) (Goldstein et al. 1995a, 1995b). Each was used with either an “ancestral haplotype” or “modal haplotype” as a root.
- Three different sets of STRs—the maximum usable set of 21 STRs (excluding only the two copies of DYS385), the same set minus the RM-YSTRs and two other loci (DYS389II and DYS448) that are potentially problematic because of complex or interrupted repeat array structure (total of 17 STRs), and finally a minimal set of 13 STRs representing the Yfiler set minus DYS385ab, DYS389II, and DYS448.
- Two different mean STR mutation rates: A slow “evolutionary” rate based on population comparisons (6.9 × 10^−^^4^/STR/generation [Zhivotovsky et al. 2004]), and a faster “pedigree” rate based on direct detection of mutations in father–son pairs (depending on the subset of STRs, 2.797–4.238 × 10^−^^3^/STR/generation; www.yhrd.org [last accessed April 17, 2014]).

**Fig. 4.**
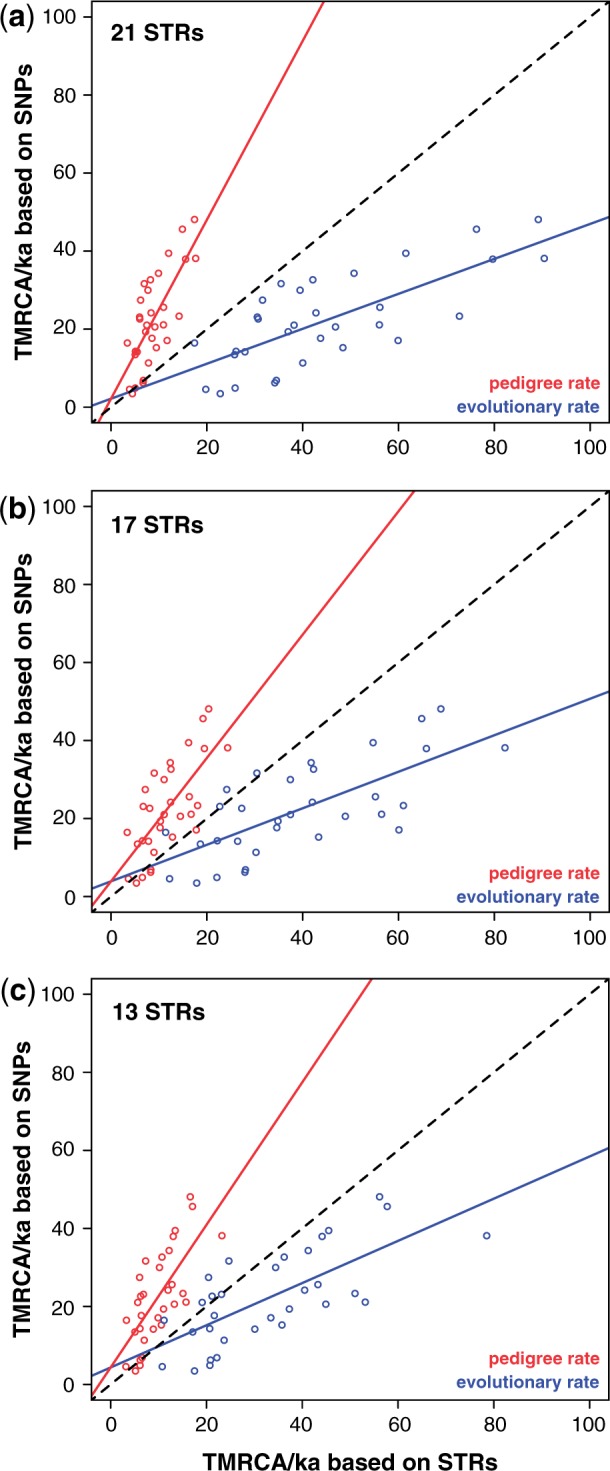
Relationship between SNP- and STR-based TMRCA estimates. SNP-based node estimates are plotted against STR-based estimates for (*a*) 21 STRs, (*b*) 17 STRs, and (*c*) 13 STRs, here using ASD with the “ancestral haplotype” root specification. The black dashed line in each case indicates *x* = *y*. Underlying data and correlation coefficients are given in supplementary tables S6 and S7, Supplementary Material online.

Supplementary table S7, Supplementary Material online, summarizes the results of comparing SNP- and STR-based TMRCA estimates for a range of nodes: Generally, the STRs perform poorly, giving a wide variety of TMRCAs for nodes with similar SNP-based dates, and correlation coefficients consistently below 0.6. Considering the variables described above: 1) ASD generally outperforms rho, and choice of rooting method (ancestral or modal) makes little difference. For rho, rooting through the ancestral haplotype performs much worse than through the modal haplotype; 2) removal of RM-YSTRs, and STRs showing repeat array complexity, does not have a major influence on relationships between SNP- and STR-based estimates of TMRCA, and the effects depend upon how the root is specified; and 3) the evolutionary STR mutation rate consistently overestimates, and the pedigree rate underestimates, the TMRCAs of nodes (fig. 4*a*). As expected, the pedigree mutation rate performs better for young nodes (<10 ka; supplementary table S6, Supplementary Material online), whereas the evolutionary rate performs better for older nodes.

### SNP Recurrence and Branch Length Heterogeneity

Of the 13,261 SNPs, 123 (0.92%) are recurrent within the tree. This is significantly lower (*P* < 0.0001, Chi square with Yates correction) than one recent study (172/5,865; 2.9% [Wei, Ayub, Chen, et al. 2013]), but significantly higher (*P* < 0.0002) than another (4/2,386; 0.2% [Scozzari et al. 2014]). Setting aside disparities in the number of individuals sequenced, these differences seem likely to be due to the sequencing strategies and the regions of the Y chromosome analyzed in the different studies. The first (Wei, Ayub, Chen, et al. 2013) is based on a whole-genome sequence data set and therefore includes repetitive elements masked in our study in which mapping may be problematic, potentially increasing the number of apparently recurrent variants. The second (Scozzari et al. 2014) is expected to reduce such mapping problems because it employs repeat-masking, as well as excluding much XY-homologous material covered in our study, in which recurrent gene conversion from the X chromosome (Rosser et al. 2009; Trombetta et al. 2010) may be active. A total of 294 events occur at the 123 recurrent sites within our tree. Of these events, 66 (22%) occur at CpG dinucleotides, where base-substitution rates are enhanced due to cytosine methylation. The remainder may include examples of XY gene conversion, and will be investigated elsewhere.

Visual inspection of the tree (fig. 3) shows apparent heterogeneity in branch lengths between (and also within) clades—for example, the tips of hg C sequences appear to extend further than those of other haplogroups. Also, one previous study has shown a significantly reduced mean number of mutations to the root for haplogroup A, compared with other lineages (Scozzari et al. 2014). One possible trivial cause of variation is tissue source for the DNAs—for example, our samples include lymphoblastoid cell-lines (LCLs) in which some somatic mutations might be expected to have accumulated (Wei, Ayub, Chen, et al. 2013), in addition to blood and buccal samples. A comparison of the mean number of mutations to the root of the tree for the three different tissue sources (supplementary fig. S3, Supplementary Material online) shows that MSY sequences in the LCLs analyzed here indeed carry significantly more mutations (mean of 471, *n* = 152; *P* = 0.00124, one-way analysis of variance) than the sequences from blood (mean of 468, *n* = 208) or saliva (mean of 466, *n* = 88). If somatic mutations are contributing to the branch lengths for LCL samples, we would expect these mutations to be found exclusively among the singleton mutations in terminal branches. The star-cluster of 44 MSY sequences found within hg R1b provides a means to test this, and given that it comprised 23 LCL and 21 non-LCL samples, has 87.5% power to detect a difference of three mutations in branch length. However, a comparison between the two sample types (supplementary table S8, Supplementary Material online) finds no significant difference (*P* = 0.73; Mann–Whitney *U* test). This apparent discrepancy may be explained by differences among the 152 analyzed LCLs in the number of passages as the cultures were first established. Considering branch lengths to the root, absolute differences between sample sources are small, so have a minimal effect on TMRCA estimates.

To address the possibility of haplogroup-specific effects, we compared the mean number of mutations with the root of our tree for 17 different major haplogroups (supplementary table S8, Supplementary Material online). Numbers of samples per haplogroup vary widely, and once this is taken into account only two comparisons, hg E and hg O versus hg R1b, retain any signal of distinctive branch lengths—for hg E, 56% of *P* values associated with Mann–Whitney *U* tests on subsampled sequences (see Materials and Methods) were significant, and for hg O the value was 87%. To ask whether this could be explained by the tissue-source effect described above, we repeated the comparisons within either LCL or non-LCL sources for these three haplogroups (supplementary table S8, Supplementary Material online). In fact, the haplogroup-specific signal is strengthened—for hg E in LCLs, 97% of *P* values associated with Mann–Whitney *U* tests on subsampled sequences were significant, and for hg O the value was 100%. For non-LCL samples, the proportion of significant *P* values in each case is 100%. We therefore conclude that subtle haplogroup-specific effects on branch length do exist.

### Putative Functional Variants and Evidence for Purifying Selection

The regions sequenced here contain 15 of the 17 single-copy MSY protein-coding genes (the missing two lie within the X-transposed region, which was not covered; fig. 1); we therefore examined variation within the coding sequences of these genes.

The 13,261 variants include 80 exonic substitutions (in 13/15 genes), of which 46 are nonsynonymous (supplementary table S9, Supplementary Material online). To assess the possible effect of natural selection on these 46 variants, we used SIFT and PolyPhen2 to predict those that were damaging to protein function, and then asked whether the proportion of singletons among damaging variants was overrepresented compared with the proportion (7,782/13,261) in the data set as a whole. For SIFT predictions, the difference is significant (17 variants, 16 of which are singletons; *P* = 0.0065). For PolyPhen2 predictions, the difference is marginally nonsignificant (15 variants, 13 of which are singletons; *P* = 0.0527); however, notably one doubleton variant is present in two Bakola samples that are sequence-identical (supplementary table S5, Supplementary Material online), and carry STR haplotypes differing by only three mutational steps at a single STR marker. This very close relationship of the two MSY sequences indicates that they have had very little time to be exposed to selective effects independently from each other. Taken together, these findings support the idea that purifying selection is acting on single-copy MSY genes.

## Discussion

The application of NGS is revolutionizing our picture of MSY diversity. Including our study, the five most recent NGS analyses summarized in figure 2 and table 1 have yielded a total of 33,479 SNPs. This tsunami of MSY variants is likely to continue, as previously unexamined populations and lineages are subjected to NGS. The 1000 Genomes Project has already contributed many more variants (Rocca et al. 2012), and a major imminent additional contribution will come from the Project’s analysis of approximately 1,250 male genomes, as well as from other sequencing projects carried out for medical genetic purposes. MSY data from these projects, like that of the Sardinian population (Francalacci et al. 2013), will be at low coverage, and therefore singleton variants will be underrepresented, so terminal branch lengths may be artificially short. Sequence capture has the advantage of high coverage and good singleton representation, but unlike the MSY data from whole-genome sequencing projects, does not come for free. Our sequence capture design yielded 3.7 Mb of usable sequence for phylogeny construction, but other designs (Poznik et al. 2013) yield more than twice as much, and indeed this appears to be the approach applied by commercial suppliers of genotyping services, which offer MSY resequencing for genealogically minded clients that will lead to many citizen-scientist generated SNPs. Currently, the nomenclature system for MSY haplogroups and variants is unstable (van Oven et al. 2014), and given all this activity there is urgent need for systematic and agreed approaches to cataloguing, validating, and naming MSY variants and lineages. In order to understand the time-depths and branching orders of different parts of the MSY phylogeny, better sampling of populations and lineages is required, and given the geographical bias of citizen-science participants this will likely be driven by academic research programs.

### MSY Mutation Rate

Although the relative ages of clades in the MSY phylogeny can now be well established thanks to the large number of variants, the absolute estimates of TMRCA remain uncertain because of corresponding uncertainty about choice of the appropriate mutation rate. Indeed recent published estimates of equivalent nodes based on NGS data vary considerably, but this is mostly ascribable to differences in assumed mutation rates. Here, we favored a rate (1.0 × 10^−^^9^/bp/year) estimated directly from NGS analysis of MSY sequences in a deep-rooting pedigree (Xue et al. 2009). Though the direct nature of the analysis and the proven transmission of newly arising variants are positive features, the study’s major disadvantage is that its mutation rate rests on only four observations. These numbers will improve as other resequencing studies are published, but meanwhile other studies (Mendez et al. 2013; Scozzari et al. 2014) have taken the genome-wide de novo mutation rate (based on a larger number of observations) and scaled it to account for male-specific transmission, thus inferring slower rates of 0.62 × 10^−^^9^ (Mendez et al. 2013) or 0.64 × 10^−^^9^ (Scozzari et al. 2014). Criticism of this approach (Elhaik et al. 2014) has been based on its indirect nature, and the fact that the resulting rates are at odds with phylogenetic mutation rate estimates (1.5–2.1 × 10^−^^9^/bp/year [Skaletsky et al. 2003; Kuroki et al. 2006]) based on human–chimpanzee MSY comparisons. Calibration based on archeological dates and assumptions about colonization history (such as the peopling of the Americas [Poznik et al. 2013] or of Sardinia [Francalacci et al. 2013]) has also been applied, although it introduces other sources of uncertainty. Further analysis of deep-rooting pedigrees, combined with accumulating data on well-dated ancient DNA, should help to give more reliable mutation rate estimates in the near future.

Visual inspection of the phylogeny suggests that there may be branch length heterogeneity within our phylogeny. However, after adjustment for sample size differences, statistical support for such differences remains for only two comparisons, hg O versus hg R1b, and hg E versus hg R1b. A truly haplogroup-specific effect of this kind would imply a *cis*-acting factor on MSY influencing mutation directly, and this seems improbable given what is known about MSY genes. A second possibility could be a factor acting over many generations in a particular geographical region or population within which a haplogroup was frequent. Such a factor could be genetic, environmental, or cultural—one possibility could be higher or lower average paternal age in a particular region, which might increase or decrease effective mutation rate for locally prevalent haplogroups. If this were the case, then we might expect haplogroups that associate together to be similarly affected: Future sequencing data on larger sample sizes should allow this to be tested.

### STR-Based TMRCA Estimation

Data presented here and elsewhere (Wei, Ayub, Xue, et al. 2013) indicate that, despite their widespread use, STRs generally perform poorly in estimating the TMRCA of haplogroups. Our expectation was that removal of STRs with particularly high mutation rates or complex internal structures might improve the performance of STR sets in TMRCA estimation. However, this was not borne out, and choice of STRs appears to make little difference. Applying the widely employed “evolutionary” STR mutation rate (Zhivotovsky et al. 2004) leads to systematic overestimation of TMRCAs compared with SNP data (though this is no longer true for all nodes if we apply a slower SNP mutation rate [Mendez et al. 2013]; supplementary table S6, Supplementary Material online); the much faster “pedigree” STR rate leads to underestimation generally, but performs better for younger clades. This probably reflects the increasing importance of back-mutation in older clades. Despite the diminishing cost of NGS, it seems likely that researchers will wish to continue to use STRs in dating; in order to provide a rational framework, careful analysis of large data sets comprising multiple STRs and MSY sequences will be needed. The “citizen-scientist” community, which now generates 111-locus STR haplotypes combined with approximately 10-Mb MSY NGS data, may be best placed to do this.

### Purifying Selection and MSY Gene Function

Our analysis of the frequency distribution of damaging variants in MSY single-copy genes suggests that purifying selection is ongoing, and that past claims of terminal degeneration of the Y chromosome are exaggerated. These findings are consistent with the picture of long-term conservation of genes from analyses of mammalian Y chromosomes (Bellott et al. 2014), as well as previous human MSY gene resequencing (Rozen et al. 2009), and general MSY sequence diversity considerations (Wilson Sayres et al. 2014). We considered only nucleotide substitutions in our analysis, and reliable indel calling is needed to provide a more thorough analysis. Although evidence is mounting that purifying selection is acting on MSY protein-coding genes, more work is required to understand their functional roles. Candidate genes are currently lacking for some established MSY-linked phenotypes such as HIV–AIDS progression (Sezgin et al. 2009) and coronary artery disease susceptibility (Charchar et al. 2012), and there is a clear need to understand the roles of noncoding RNA genes on the MSY, as well as the suite of protein-coding genes.

### Molecular Evolutionary Applications of High-Resolution MSY Phylogenies

Previously, we and others have taken a phylogenetic approach to analyzing the mutational history of other classes of Y-chromosomal variants, including structural rearrangements (Repping et al. 2006; Jobling et al. 2007; Balaresque, Bowden, et al. 2008; Balaresque, Parkin, et al. 2008), intrachromosomal gene-conversion events (Rozen et al. 2003; Bosch et al. 2004; Hallast et al. 2013; Balaresque et al. 2014), and gene-conversion between the X and Y chromosomes outside the pseudoautosomal regions (Rosser et al. 2009; Trombetta et al. 2010, 2014). Such analyses require both a reliable phylogeny and a means of assaying the complex variants. NGS can now provide phylogenies of very high resolution, in which almost all males in a sample can be distinguished, and the phylogenetic and temporal relationships between their MSY sequences can be described in a fine-grained and unbiased way. In principle, NGS can simultaneously identify the associated complex variants. However, using NGS data to unambiguously determine the allelic states of variants in highly similar regions within the MSY, and between the MSY and the X chromosome, is challenging. Overcoming these difficulties will lead to unprecedented illumination of the complex mutational history of the Y chromosome.

## Materials and Methods

### Samples

DNA donors were recruited with informed consent. Human DNAs (supplementary material and table S1, Supplementary Material online) were extracted from saliva (using the Oragene kit), LCLs, or peripheral blood. Twenty males from each of 19 populations were supplemented by 77 assorted samples chosen based on prior Y haplogroup information. Four HapMap populations (CEU [Utah Residents (CEPH) with Northern and Western European ancestry], TSI [Toscani in Italia], YRI, and CHB) were included, both as part of the population data set, and to provide data on externally analyzed samples for validation purposes. Two non-HapMap individuals were subsequently identified as females and removed from all downstream analysis, reducing the final number of sequenced males to 455.

### Sequencing, Data Analysis, Variant Calling, and Filtering

For details of all procedures, see supplementary material, Supplementary Material online. 

Briefly, 3–5 µg of genomic DNA was used for library preparation and target enrichment, followed by paired-end 100-bp Illumina sequencing. Reads were mapped to the human genome reference (GRCh37), followed by local realignment, duplicate read marking, and base quality score recalibration.

Variant calling and filtering were carried out leading to a final analyzed region of 3,724,156 bp. In total 19,276 raw variants were called from 455 samples, and following filtering 13,261 sites in 448 samples were retained for all downstream analyses. In silico validation was done using Complete Genomics whole-genome sequence data (8 samples) and Omni2.5 BeadChip genotype data (88 samples). Based on the Complete Genomics comparison, the false-positive error rate was 0% and false-negative error rate 0.009%; more details, including genotype-based error rates, are given in supplementary material and table S10, Supplementary Material online. All variants have been submitted to dbSNP (supplementary table S4, Supplementary Material online), and a vcf is available from https://www2.le.ac.uk/departments/genetics/people/jobling/publications.

### Phylogenetic Inference and Dating

Maximum-parsimony trees were created in PHYLIP v3.69 (Felsenstein 2005) and visualized using FigTree v1.4.0 (Rambaut 2006–2012). Ancestral states were defined using information from the phylogeny and from sequence data from 22 male great apes, generated concurrently with the human sequencing (supplementary material, Supplementary Material online). For assignment of variants to branches, see supplementary table S11 and figure S4, Supplementary Material online.

TMRCA and its standard deviation were estimated for clades within the PHYLIP outfile using the rho statistic (Forster et al. 1996; Saillard et al. 2000) implemented in a Perl script. A scaled rate of one mutation per 268.5 years was used, based on 1.0 × 10^−^^9^ mutations/nucleotide/year (Xue et al. 2009) and the number of nucleotides in our regions of interest (3,724,156). We assumed a generation time of 30 years. In addition, to capture the uncertainty in the published mutation rate we calculated TMRCA based on the bounds of its 95% confidence interval: 3.0 × 10^−^^10^–2.5 × 10^−^^9^ mutations/nucleotide/year (Xue et al. 2009).

Known Y-SNPs were sought using AMY-tree v1.2 (Van Geystelen et al. 2013a, 2013b), and v2.0 of the same software (with the option of specifying MSY subregions) was used for variant validation through phylogenetic consistency.

### Y-STR Analysis

In total, 23 Y-STRs (DYS19, DYS389I, DYS389II, DYS390, DYS391, DYS392, DYS393, DYS385ab, DYS437, DYS438, DYS439, DYS448, DYS456, DYS458, DYS635, GATAH4, DYS481, DYS533, DYS549, DYS570, DYS576, and DYS643) were typed in all samples using the PowerPlex Y23 system (Promega) according to manufacturer’s instructions. TMRCA was calculated using 21 STRs (omitting the bilocal DYS385ab), 17 STRs (additionally omitting the RM-YSTRs DYS570, DYS576 and the complex STRs DYS389II, DYS448), or 13 STRs (additionally omitting the non-Yfiler loci DYS481, DYS533, DYS549, DYS643). Dating methods were rho, implemented within the program NETWORK 4.612 (Bandelt et al. 1999), and ASD (Goldstein et al. 1995a, 1995b). Further details are given in supplementary material, Supplementary Material online.

### Branch Length Heterogeneity Testing

Differences in branch length across the 17 haplogroups were assessed with a pairwise comparison using a Mann–Whitney *U* test. Bonferroni correction was also applied to account for multiple pairwise tests. For haplogroups with *n* > 10, ten random individuals were sampled to account for sample size variation. This process was repeated 100 times producing 100 *P* values for each comparison. The proportion of significant *P* values was then calculated and only comparisons with a proportion greater than 50% were considered of interest.

### Genes and Functional Variants

Variants within UCSC genes within the sequenced regions were identified and their likely functional effects analyzed using wANNOVAR (Chang and Wang 2012).

## Supplementary Material

Supplementary material, tables S1–S12, and figures S1–S5 are available at *Molecular Biology and Evolution* online (http://www.mbe.oxfordjournals.org/).

Supplementary Data
