# Supplementary material for: The Y-Chromosome Tree Bursts into Leaf: 13,000 High-Confidence SNPs Covering the Majority of Known Clades
Source: Mol Biol Evol. 2014 Dec 2;32(3):661–73. doi: 10.1093/molbev/msu327 (PMC4327154; doi:10.1093/molbev/msu327)
Supplement: Supplementary Data [file supp_32_3_661__index.html]

The Y-Chromosome Tree Bursts into Leaf: 13,000 High-Confidence SNPs Covering the Majority of Known Clades — The Y-Chromosome Tree Bursts into Leaf: 13,000 High-Confidence SNPs Covering the Majority of Known Clades — Supplementary Data 

# The Y-Chromosome Tree Bursts into Leaf: 13,000 High-Confidence SNPs Covering the Majority of Known Clades

## Supplementary Data

files

**Files in this Data Supplement:**

- Supplementary Data - pdf file
- Supplementary Data - pdf file
- Supplementary Data - pdf file
- Supplementary Data - txt file
- Supplementary Data - xls file
- Supplementary Data - xls file
- Supplementary Data - docx file
