# Supplementary material for: The Y-Chromosome Tree Bursts into Leaf: 13,000 High-Confidence SNPs Covering the Majority of Known Clades
Source: Mol Biol Evol. 2014 Dec 2;32(3):661–73. doi: 10.1093/molbev/msu327 (PMC4327154; doi:10.1093/molbev/msu327)
Supplement: Supplementary Data [file supp_msu327_FigureS1_TreeWithSampleNames.pdf]

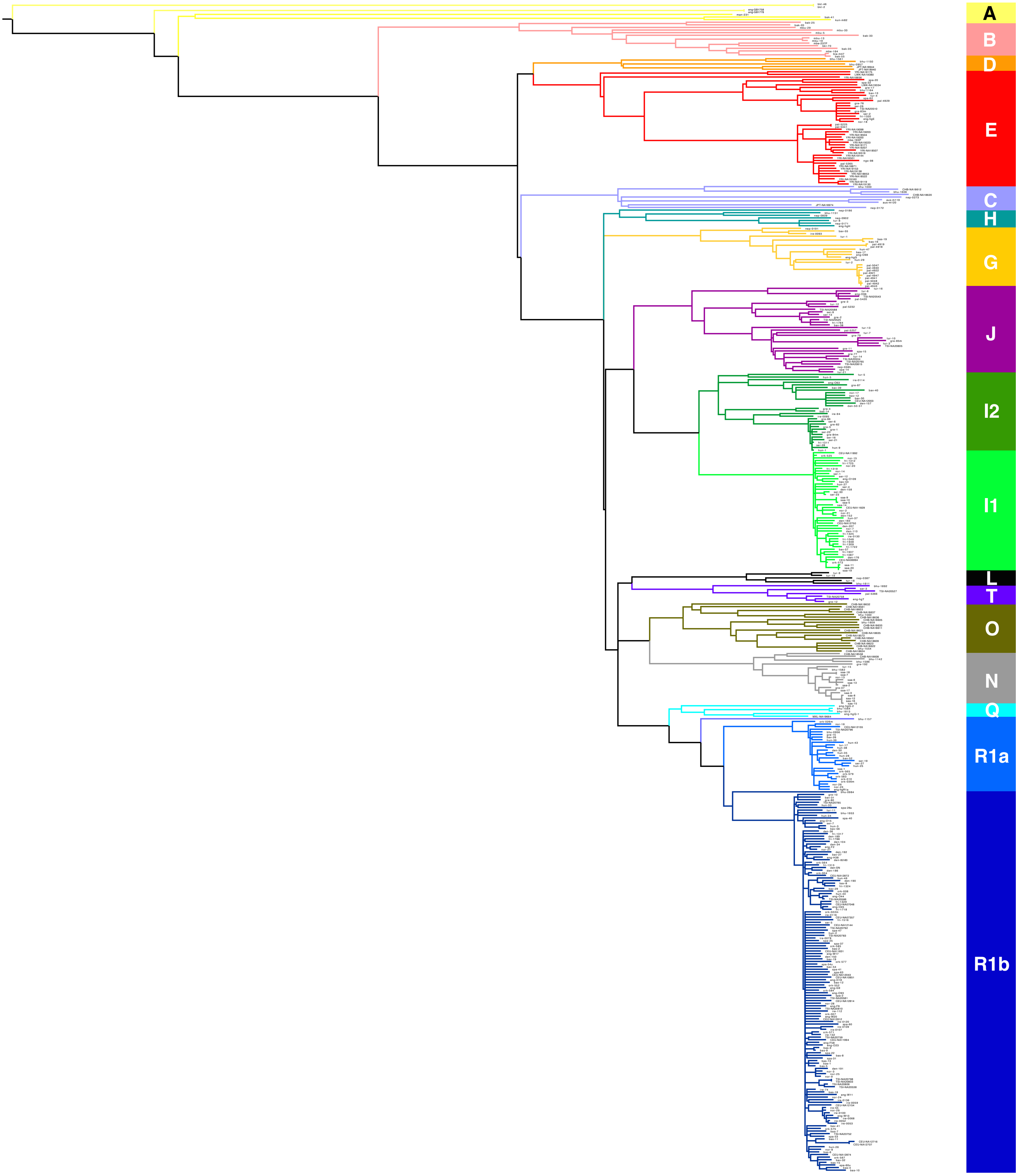

Figure S1: Maximum-parsimony tree showing sample names.
The phylogeny is the same as that shown in Figure 3, but includes sample names.
