# Supplementary material for: The Y-Chromosome Tree Bursts into Leaf: 13,000 High-Confidence SNPs Covering the Majority of Known Clades
Source: Mol Biol Evol. 2014 Dec 2;32(3):661–73. doi: 10.1093/molbev/msu327 (PMC4327154; doi:10.1093/molbev/msu327)
Supplement: Supplementary Data [file supp_msu327_FigureS2_Networks.pdf]

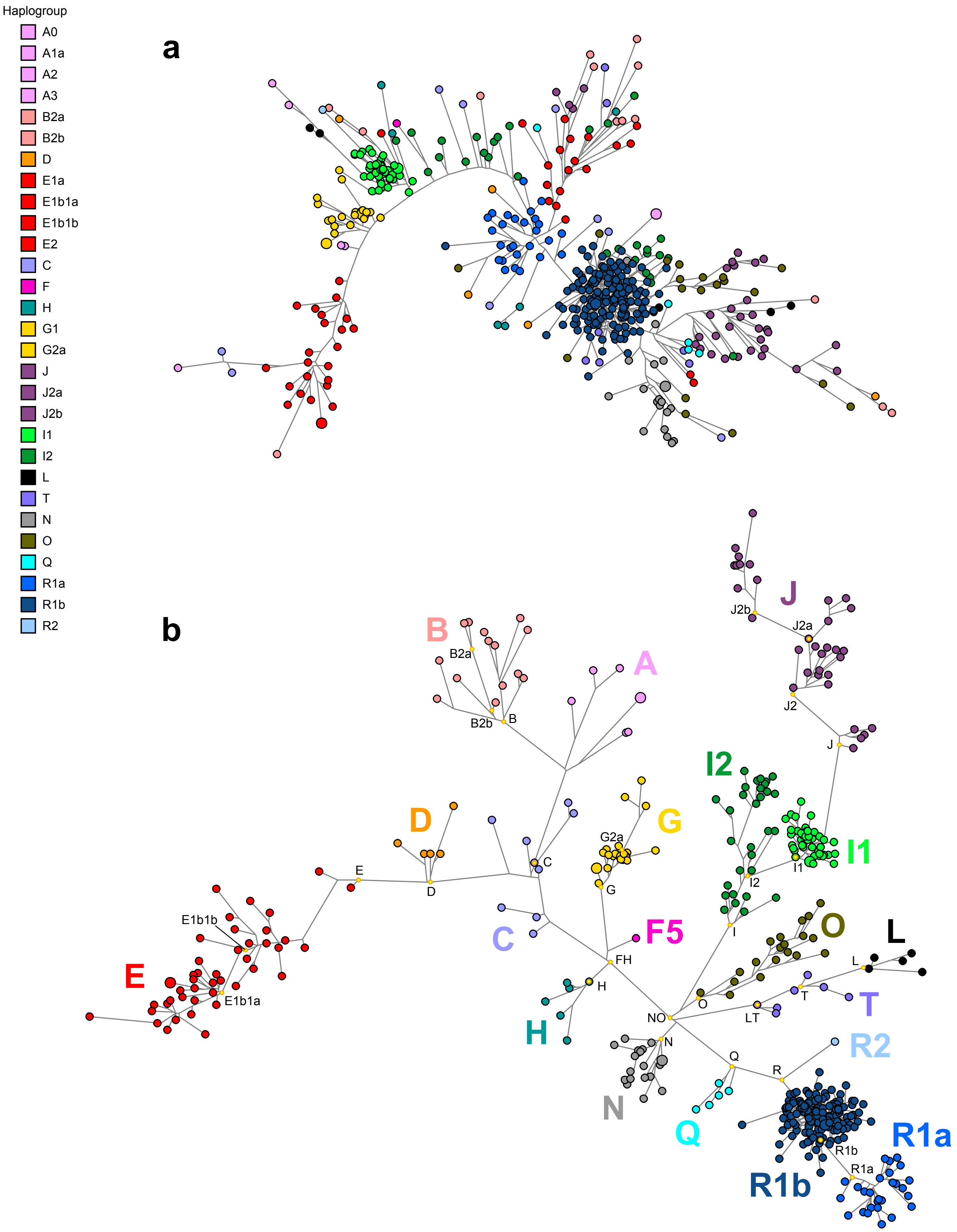

Figure S2: Median-joining networks of STR haplotypes.
Networks constructed from 21 Y-STRs (Powerplex Y23 set minus DYS385a,b – see main text). Circles represent haplotypes, with area proportional to sample size, and lines between them mutational steps. Colours represent haplogroups given in the key to the left. (a) Network constructed from STR data alone. Note the six shared haplotypes, and the fact that young clades (e.g. hgI1) tend to form discrete clusters, while older clades (e.g. hgI2) are dispersed. (b) Network constructed from STR data plus 5 branch-defining SNPs per haplogroup to enforce the phylogenetic structure. Small yellow circles indicate nodes used for 'ancestral haplotype' dating, and are labelled with (sub)haplogroup names.
