# Supplementary material for: The Y-Chromosome Tree Bursts into Leaf: 13,000 High-Confidence SNPs Covering the Majority of Known Clades
Source: Mol Biol Evol. 2014 Dec 2;32(3):661–73. doi: 10.1093/molbev/msu327 (PMC4327154; doi:10.1093/molbev/msu327)
Supplement: Supplementary Data [file supp_msu327_FigureS3_TissueSourse_BranchLength.pdf]

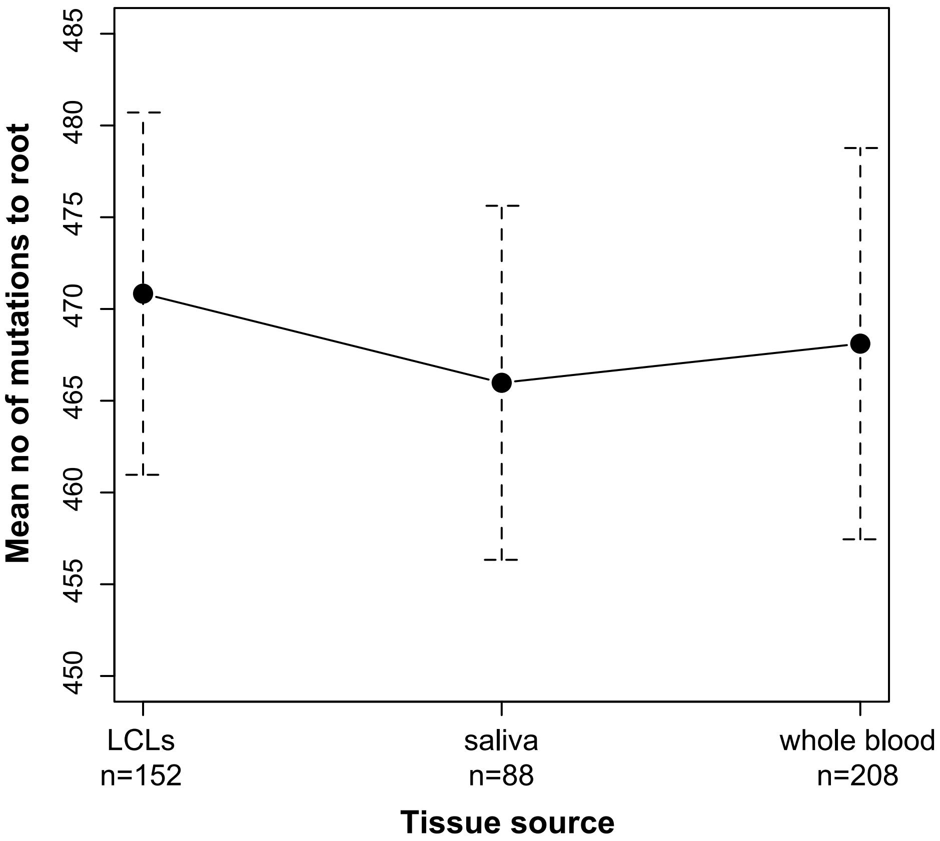

Figure S3: Influence of different tissue sources on mutational branch lengths.
Bars indicate standard deviations. LCLs: lymphoblastoid cell-lines.
