# Supplementary material for: The Y-Chromosome Tree Bursts into Leaf: 13,000 High-Confidence SNPs Covering the Majority of Known Clades
Source: Mol Biol Evol. 2014 Dec 2;32(3):661–73. doi: 10.1093/molbev/msu327 (PMC4327154; doi:10.1093/molbev/msu327)
Supplement: Supplementary Data [file supp_msu327_YphyloTreePaperSuppInfoRevised_281014.docx]

**Supplementary Material**

**Materials and Methods**

***Samples***

The total number of sequenced individuals was 455, made up of 378 males recruited on a population basis, plus 77 males from various populations chosen based on prior haplogroup information. For further details on samples see Table S1. DNA donors were recruited with informed consent (research ethics reference number: maj4-cb66). Human DNA samples were extracted from various sources including lymphoblastoid cell lines, peripheral blood, and saliva.

For the population sample, a total of 380 individuals (later reduced to 378) were included in the design from 19 populations (20 males each) mostly from Europe and the Near East, but including one population each from Africa and East Asia. Samples from Greece, Serbia, Hungary, Germany [Bavaria], Spanish Basque country, central Spain, Netherlands [Frisia], Denmark, Norway, Finland [Saami], England [Herefordshire and Worcestershire] ([Winney et al. 2012](#_ENREF_20)), Orkney ([Winney et al. 2012](#_ENREF_20)), Ireland and Turkey were collected by the authors. Twenty random Palestinian male samples were purchased from the National Laboratory for the Genetics of Israeli Populations ([www.tau.ac.il/medicine/NLGIP)](http://www.tau.ac.il/medicine/NLGIP),a). Finally, samples from four HapMap ([International HapMap Consortium 2010](#_ENREF_11)) populations were used, both to supplement the population dataset, and to provide data on externally analysed samples for validation purposes: from Europe, the Centre d'Etude du Polymorphisme Humain (CEPH) collection in Utah, USA, with ancestry from Northern and Western Europe (CEU) and the Toscani in Italia (TSI); from Africa, the Yoruba from Ibadan, Nigeria (YRI); and from China, the Han Chinese from Beijing (CHB). After the initial analyses, one English and one Spanish sample were identified as females and therefore removed from all downstream analyses in this study.

***Bait design for target enrichment***

Agilent SureSelect (Agilent Technologies Inc., CA, USA) hybridisation capture was used for target enrichment. RNA baits were designed using Agilent eArray using default parameters for Illumina Paired-End Long Read sequencing (Bait length: 120 nt; Design strategy: centered; Tiling frequency: 1X; Avoid overlap: 20 bp; Strand: sense; Avoid regions: Repeat Masker) and human reference sequence hg19/GRCh37 (February 2009). Boosting was used for ‘orphan’ (located >20 bp from flanking baits) and GC-rich (≥63%) baits by direct replication (orphans 2 ×, GC-rich 3 ×).

Baits covered a total of 10.9 Mb, including some autosomal and X-specific sequences, and Y-chromosome sequences including segments of palindromic repeats, part of the X-transposed region, and pseudoautosomal genes, that will be described elsewhere. In this study we focus on the 8 X-degenerate regions ([Skaletsky et al. 2003](#_ENREF_16)) which are likely to yield interpretable sequence data in which variants can be sought. The total length of these targeted regions was ~8.6 Mb, and following capture design and the necessary repeat masking, the 19,209 baits designed covered 2.3 Mb. Coordinates of the 8 targeted regions can be found in Table S2.

***Sequencing and data processing***

3-5 µg of genomic DNA was used for library preparation and target enrichment using Agilent SureSelect^XT^ Target Enrichment System for Illumina Paired-End Sequencing Library kit (Version 1.3) according to manufacturer’s recommendations with some modifications. DNA samples were fragmented to ~250-600 bp without size selection, to obtain larger insert sizes. These changes to the original protocol resulted in a mean insert size of 330 bp, thus increasing recovery of sequence data from bait-adjacent regions. Sequencing was done on an Illumina HiSeq 2000 instrument (Illumina, CA, USA) with paired-end 100-bp run. Library preparation, target enrichment and sequencing were done at the Oxford Genomics Centre at the Wellcome Trust Centre for Human Genetics, University of Oxford, UK.

Base calling was done using Illumina Bustard ([Kao et al. 2009](#_ENREF_12)) and quality control with FastQC ([Andrews 2012](#_ENREF_2)). Reads were mapped to the human genome reference (GRCh37) using Stampy v1.0.20 ([Lunter and Goodson 2011](#_ENREF_14)). Local realignment was done using The Genome Analysis Toolkit (GATK) v2.6-5 ([DePristo et al. 2011](#_ENREF_6)), followed by duplicate read marking with Picard v1.86 ([Wysoker et al. 2009](#_ENREF_21)) and base quality score recalibration with GATK. The individual steps and parameters used are listed in Table S12.

***Data analysis, variant calling and filtering***

Increased insert sizes (see above) and efficient sequence capture led to high sequence coverage not only at baited regions but also at ~300 bp regions flanking the baits. The original bait coordinates were therefore extended by 300 bp at each end, and overlapping coordinates merged. This increased the analysed region to 4,433,580 bp.

The samtools mpileup v0.1.19 multi-sample option was used for variant calling, treating all samples simultaneously with the following general parameters: minimum base quality 20, minimum mapping quality 50 and no INDEL calling (Table S12). In total 19,276 raw SNPs were called from 455 males.

Raw variants were filtered using vcftools v0.1.11 ([Danecek et al. 2011](#_ENREF_5)) and in-house Perl scripts (Table S12). Filtering was based on clustered SNPs (reducing the number of variants to 18,200); strand bias, minimum depth 6 ×, removal of heterozygous calls and sites with ≥5% missing calls (reducing variants to 13,543), followed by removal of samples with ≥5% missing calls and invariable sites, resulting in a final dataset of 13,474 sites.

Seven male samples were missing more than 5% of genotypes due to low coverage, contamination and an interstitial deletion (Table S1), and therefore were removed from the final filtered dataset, which included 13,474 variant sites from 448 samples, with 0 - 643 missing genotypes per individual, and a 99.8% average call rate.

In order to circumvent the problem of missing genotypes for subsequent analyses, we attempted to recover as many as possible. Missing genotypes were divided into three groups based on read-depth: DP 0 - the genotype call was discarded; DP 2-6 - the raw call was accepted; all other cases - the sites were re-called using a single-sample approach in order to obtain the DP4 field in the vcf, the bam file was checked manually, and the most probable allele was inferred by comparing the bam file with the information contained in the DP4 field. After this procedure, 213/13,474 sites still lacked genotype calls, leading to a final number of 13,261 sites for further analyses.

Having applied the above filters to variant sites, it was necessary to apply the same criteria to non-variant sites in order to provide an accurate ratio between the two, important for estimating TMRCA, for example. We calculated the depth per sample per site using the GATK DepthOfCoverage tool, filtered for base quality 20 and mapping quality 50, and then applied the criterion of ≥6 × coverage in ≥95% of samples. This procedure led to a reduction in the figure of base-pairs sequenced from 4,433,580 bp to 3,724,156 bp. The corresponding coordinates (Table S3) were used for all downstream analysis.

Mean raw sequence coverage per sample across the 3,724,156 bp of analysed regions was calculated using Picard v1.93. Sequence depth for the 448 samples varied from 25 × to 85 × per sample, with the average of 51 × (Table S1).

***Validation***

*In silico* validation of the 13,261 filtered SNP calls was done using two previously published data sets: genomes sequenced to high-coverage with self-assembling DNA nanoarrays by Complete Genomics ([Drmanac et al. 2010](#_ENREF_7)), and Omni2.5 BeadChip genotype data produced at the Broad Institute as part of the 1000 Genomes Project ([1000 Genomes Project Consortium 2012](#_ENREF_1)). Our 455 samples included 8 and 88 individuals from the HapMap collection overlapping with the Complete Genomics and Omni genotype data, respectively. A Perl script was written to compare the SNP calls in our variant set to overlapping samples and positions in the control sets.

Of the 1365 variant sites shared between our data and the Complete Genomics data across 8 overlapping samples, the false-positive error rate was 0% and false-negative error rate 0.009%. When compared to the Omni data across 298 variant sites and 88 overlapping samples, the error rates were higher: 0.68% for false positives and 2.30% for false negatives. However, all the false calls originated from only 33 variant sites.

In order to shed light on these comparatively high error rates, we also compared Complete Genomics and Omni data for regions corresponding to our final analyzed regions. Across 263 variant sites and 49 overlapping samples, we obtained false-positive and false-negative rates of 2.85% and 2.36%, respectively. The false calls originated from 30 sites and 18 of those overlap with the sites producing high error rates when comparing our data with Omni. Since the Complete Genomics dataset is generally considered to have very high quality then this seems to indicate problems in making correct calls from Omni genotyping data.

Results are given in more detail in Table S10.

***Phylogenetic Inference***

PHYLIP v3.69 was used to create a maximum parsimony phylogenetic tree ([Felsenstein 2005](#_ENREF_8)). Three independent trees were constructed using randomisation of input order (seeds: 4941985, 62529981 and 38185313), each 10 times. Output trees of these runs were used to build a consensus tree with the consense program included in PHYLIP package.

The tree was rooted using the chimpanzee MSY reference sequence. FigTree v1.4.0 ([Rambaut 2006-2012](#_ENREF_15)) was used to visualise the tree (tree.bio.ed.ac.uk/software/figtree/).

***Ancestral states of variants***

In order to define the ancestral states for the final filtered variants we used the phylogenetic information from the maximum parsimony tree of human samples extracted from the PHYLIP outfile, as well as sequencing data generated from a number of great ape males.

The following great ape samples were sequenced using an identical approach to the human samples: 8 common chimpanzees (*Pan troglodytes*), 3 bonobos (*Pan paniscus*), 6 gorillas (*Gorilla gorilla*), 5 orangutans (2 Bornean orangutans [*Pongo pygmaeus*], and 3 Sumatran orangutans [*Pongo abelii*]). Sequence data processing was identical to human samples and due to the absence of Y-chromosome reference sequence for gorilla and orangutan, the human reference sequence was used to map the data from all species. 13,261 final human variants were called with samtools mpileup multi-sample option per species using the same parameters as for human samples (Table S12). Data were filtered for strand bias (SP>13) and minimum depth ≥ 1, and the nucleotide state for each species was defined only if it was supported by at least two individuals.

If an allelic state for a human variant matched the ortholog in the chimpanzee and/or bonobo data, this was defined as the ancestral state. If human alleles did not match with either *Pan* species, the procedure was repeated for the gorilla sequence, and if gorilla did not match then the orangutan sequence was analysed.

For 12,798 sites the allelic state defined using ape sequences matched with the reconstructed human ancestral state from the PHYLIP output. For 157 sites where the ape data were missing or did not match either human allele, the allelic state from the deepest rooting branch (A0) of the human phylogenetic tree was used. For 295 sites the allele from apes matched with a singleton in human data or an allele specific to a distal branch in the tree. For those cases the allelic state of human deep-rooting branches was assumed to be the ancestral state for all humans. After applying these criteria an additional 11 sites were left and for those the ancestral allele was assigned randomly.

***Haplogroup prediction, and variant validation via phylogenetic consistency***

The presence of known Y-chromosome markers was checked using AMY-tree v1.2 ([Van Geystelen et al. 2013a](#_ENREF_17); [Van Geystelen et al. 2013b](#_ENREF_18)). This software was developed to check if the allelic states of known Y-chromosome markers are consistent with the Y Chromosome Consortium phylogenetic tree ([Karafet et al. 2008](#_ENREF_13)) and its subsequent updates, and to predict the haplogroup using whole-genome sequence data. Concordance with the expected phylogeny and therefore the quality of the SNPs calls is given as a MCC (Matthews correlation coefficient) value per sample. Samples with MCC values ≥0.95 are considered to have excellent SNP calling quality, meaning that ≥97.5% of negative and positive allelic predictions are concordant with the expected states of the phylogenetic tree. Since our data do not cover the whole Y chromosome but only a proportion of it, v1.2 of the software lacked sufficient information for haplogroup prediction. However, we used it to deduce the presence and allelic states of known Y-markers present in sequence data and for assigning a standard haplogroup to all our samples (Table S1).

A modified version of AMY-tree v2.0 (available at <http://bio.kuleuven.be/eeb/lbeg/software>) was developed that can use user-defined regions rather than whole Y chromosome data. This was used as an additional validation to check for phylogenetic consistency of our data with v2.2 MutationConversion and UpdatedTree files and to obtain MCC values based on the specific regions sequenced. The mean MCC value for our samples is 0.969 (range from 0.887 to 1, median 0.968), confirming very high quality of our data both in covering the expected known markers in the sequenced regions, but also high concordance of the calls with the expected phylogenetic tree.

A total of 55/1152 sites were marked as sources of false calls based on AMY-tree analysis; however, 51 of these, expected by the program to be present in specific allelic states in specific branches, had been discarded from our data in the filtering stage. These 51 sites are therefore not real false calls, and if they were ignored by AMY-tree, then the MCC values for majority of our samples would be 1, meaning 100% concordance of the allelic states of all known markers in the sequenced regions with the expected phylogenetic tree. The remaining four sites were manually checked in bam files:

• PK2 (hgC3, T>C) has the ancestral allele in all our samples, including the four samples defined as C3 (bhu-1000, bhu-1606, CHB-NA18612 and CHB-NA18620). Read depth at this position varies from 42 × to 73 ×, all reads supporting the ancestral allele. bhu-1000 carries the derived alleles for M48, M77, and M86, and therefore on the basis of a published tree ([Karafet et al. 2008](#_ENREF_13)) and the one which was used in AMY-tree [Van Geystelen, 2014 #3464], this sample at least is expected to carry the derived allele at PK2. This indicates, as suggested on the relevant ISOGG (version 9.76) tree page (<http://www.isogg.org/tree/ISOGG_HapgrpC.html>) that PK2’s position is uncertain. Therefore, it is recommended to exclude marker PK2 from the currently used phylogeny in AMY-tree.

• M165 (hgE1b1b1b, A>G) is expected by AMY-tree to be derived in our sample spa-32 because this sample carries the marker M183 in its derived state; however, all 105 reads covering this position in the specified sample have the ancestral allele. According to the relevant ISOGG (version 9.76) tree page (<http://www.isogg.org/tree/ISOGG_HapgrpE.html>), M165 is a terminal marker, downstream of M183, so the derived state of the latter cannot be used to predict the allelic state of the former. The M165 call is therefore not incorrect, and a correction in the currently used phylogeny in AMY-tree will be made.

• L474 (hgQ1a, C>T) is expected to have the derived allele in four of our samples according to the AMY-tree phylogeny [Van Geystelen, 2014 #3464] and to the ISOGG (version 9.76) tree (http://www.isogg.org/tree/ISOGG_HapgrpQ.html) since they carry markers that are phylogenetically more derived. Consistent with this, our samples eng-hgQ-1 and MXL-NA19664 (hgQ1a3a-M3) indeed have the derived allele, but our two samples bhu-1564 and bhu-1813 have the ancestral allele, with read depths of 50 × and 52 × respectively at the site, despite carrying the derived allele at M120. This suggests either that the phylogenetic position of the variant is incorrect, or recurrence of L474. We note that the site does not lie in a region of high gametology, nor at a CpG dinucleotide. Analysis of additional samples assigned to hgQ1 is required to deal with this issue.

• The final site (P134) has the unexpected allele in only one sample, and has been identified as a recurrent mutation in our tree. The variant lies in a region of high similarity (gametology) with the X chromosome (mean 94% over the surrounding 120 bp), and the non-reference allele is identical to the X sequence state. Together with the high local read-depth (Y: 128 ×; X: 139 ×), this strongly suggests that this variant is real, and likely derives from an X-to-Y gene conversion event.

Taken together, these considerations indicate that all called variants tested by AMY-tree are true.

***STR data analysis***

23 Y-STRs (DYS19, DYS389I, DYS389II, DYS390, DYS391, DYS392, DYS393, DYS385ab, DYS437, DYS438, DYS439, DYS448, DYS456, DYS458, DYS635, GATAH4, DYS481, DYS533, DYS549, DYS570, DYS576, and DYS643) were typed using the PowerPlex® Y23 system (Promega). TMRCA was calculated using 21 STRs (omitting the bilocal DYS385ab), 17 STRs (additionally omitting the RM-YSTRs DYS570, DYS576 and the complex STRs DYS389II, DYS448), or 13 STRs (additionally omitting the non- Yfiler® loci DYS481, DYS533, DYS549, DYS643). Median-joining networks were constructed in the program NETWORK 4.612, with STRs weighted as previously ([Batini et al. 2011](#_ENREF_4)). Partial alleles were rounded up to the next largest integral alleles, and duplicated alleles also considered only the larger allele. SNPs were included in the network (Figure S2b) to enforce a coherent phylogenetic structure – 5 were included on each of 52 branches, with weighting of 49-99 depending to on the hierarchical position in the tree ([Batini et al. 2011](#_ENREF_4)). Dating methods were rho, implemented within NETWORK 4.612 ([Bandelt et al. 1999](#_ENREF_3)), and average squared distance ([Goldstein et al. 1995a](#_ENREF_9); [Goldstein et al. 1995b](#_ENREF_10)). Both methods require the specification of a root from which to count the number of mutations accumulated by a set of haplotypes, and this was assigned to a putative ‘ancestral haplotype’ (or median vector) within the network (Figure S2b). In addition, a synthetic ‘modal haplotype’ combining the modal values for individual STR repeat counts (ASD), or the real haplotype in the network closest to this modal haplotype (rho) was used. Calculations used either a uniform mean evolutionary rate of 6.9 x 10^-4^ per STR per generation ([Zhivotovsky et al. 2004](#_ENREF_22)), or a mean pedigree rate based on data available at [www.yhrd.org](http://www.yhrd.org), with means of the associated 95% confidence intervals. For 21 STRs the pedigree rate was 4.238 (2.1928-8.654) x 10^-3^, for 17 it was 2.797 (1.567-6.105) x 10^-3^, and for 13 it was 2.797 (1.933-3.944) x 10^-3^ per locus per generation.

We chose not to use the coalescent dating method implemented in BATWING ([Wilson and Balding 1998](#_ENREF_19)) because our dataset does not fit the necessary population assumptions.

**References**

1000 Genomes Project Consortium. 2012. An integrated map of genetic variation from 1,092 human genomes. Nature 491:56-65.

Andrews, S. 2012. FastQC, <http://www.bioinformatics.babraham.ac.uk/projects/fastqc/>.

Bandelt, H-J, P Forster, A Röhl. 1999. Median-joining networks for inferring intraspecific phylogenies. Mol Biol Evol 16:37-48.

Batini, C, G Ferri, G Destro-Bisol, et al. 2011. Signatures of the pre-agricultural peopling processes in sub-Saharan Africa as revealed by the phylogeography of early Y chromosome lineages. Mol Biol Evol 28:2603-2613.

Danecek, P, A Auton, G Abecasis, et al. 2011. The variant call format and VCFtools. Bioinformatics 27:2156-2158.

DePristo, MA, E Banks, R Poplin, et al. 2011. A framework for variation discovery and genotyping using next-generation DNA sequencing data. Nat Genet 43:491-498.

Drmanac, R, AB Sparks, MJ Callow, et al. 2010. Human genome sequencing using unchained base reads on self-assembling DNA nanoarrays. Science 327:78-81.

Felsenstein, J. 2005. PHYLIP (Phylogeny Inference Package) version 3.6. Distributed by the author (Department of Genome Sciences, University of Washington, Seattle, WA).

Goldstein, DB, AR Linares, LL Cavalli-Sforza, MW Feldman. 1995a. An evaluation of genetic distances for use with microsatellite loci. Genetics 139:463-471.

Goldstein, DB, AR Linares, LL Cavalli-Sforza, MW Feldman. 1995b. Genetic absolute dating based on microsatellites and the origin of modern humans. Proc Natl Acad Sci USA 92:6723-6727.

International HapMap Consortium. 2010. Integrating common and rare genetic variation in diverse human populations. Nature 467:52-58.

Kao, WC, K Stevens, YS Song. 2009. BayesCall: A model-based base-calling algorithm for high-throughput short-read sequencing. Genome Res 19:1884-1895.

Karafet, TM, FL Mendez, M Meilerman, PA Underhill, SL Zegura, MF Hammer. 2008. New binary polymorphisms reshape and increase resolution of the human Y-chromosomal haplogroup tree. Genome Res 18:830-838.

Lunter, G, M Goodson. 2011. Stampy: a statistical algorithm for sensitive and fast mapping of Illumina sequence reads. Genome Res 21:936-939.

Rambaut, A. 2006-2012. Fig.Tree. Tree Figure Drawing Tool, version 1.4.0. Available at: <http://tree.bio.ed.ac.uk/software/figtree/>.

Skaletsky, H, T Kuroda-Kawaguchi, PJ Minx, et al. 2003. The male-specific region of the human Y chromosome: a mosaic of discrete sequence classes. Nature 423:825-837.

Van Geystelen, A, R Decorte, MH Larmuseau. 2013a. AMY-tree: an algorithm to use whole genome SNP calling for Y chromosomal phylogenetic applications. BMC Genomics 14:101.

Van Geystelen, A, R Decorte, MH Larmuseau. 2013b. Updating the Y-chromosomal phylogenetic tree for forensic applications based on whole genome SNPs. Forensic Sci Int Genet 7:573-580.

Wilson, IJ, DJ Balding. 1998. Genealogical inference from microsatellite data. Genetics 150:499-510.

Winney, B, A Boumertit, T Day, et al. 2012. People of the British Isles: preliminary analysis of genotypes and surnames in a UK-control population. Eur J Hum Genet 20:203-210.

Wysoker, A, K Tibbetts, T Fennell. 2009. Picard v1.86, available from <http://picard.sourceforge.net/>.

Zhivotovsky, LA, PA Underhill, C Cinnioglu, et al. 2004. The effective mutation rate at Y chromosome short tandem repeats, with application to human population-divergence time. Am J Hum Genet 74:50-61.

**Table S12. Software tools and parameters used in data analysis.**

| **Function** | **Tool** | **Package** | **Parameters** |
| --- | --- | --- | --- |
| Call bases | Illumina Bustard | |  |
| Quality control | FastQC | |  |
| Map reads | Stampy v1.0.20 | | -g GRCh37 -h GRCh37 --fast --insertsize=330 --insertsd=120 –xa-max=2 --xa-max-discordant=2 |
| Local realignment | RealignerTargetCreator | GATK v2.6-5 | -R GRCh37 |
|  | IndelRealigner |  | -R GRCh37 -targetIntervals file_made_by_RealignerTargetCreator |
| Duplicate marking | MarkDuplicates | Picard v1.86 | N/A |
| Base quality score recalibration | BaseRecalibrator | GATK v2.6-5 | -R GRCh37 --covariate CycleCovariate --covariate ContextCovariate -knownSites broad_dbsnp -knownSites known_phase1_indels -knownSites known_mills_indels  (plus ReadGroupCovariate and QualityScoreCovariate by default) |
| Raw sequence depth | CalculateHsMetrics | Picard v1.93 | BAIT_INTERVALS= analysed_coordinate_file TARGET_INTERVALS=original_8target_intervals |
| SNP calling | mpileup | SAMtools v0.1.19 | -Q 20 -q 50 -I -D -S -g -O -s -f B37 -l analysed_coordinate_file |
|  | bcftools view |  | -v -c -g |
| Filtering | Vcftools v0.1.11 and Perl scripts | | Maximum 3x average DP per site across all samples  SnpCluster [2,4]  Strand bias (SP>13)  Minimum DP 6  Remove heterozygous calls  Maximum missing data per site – 5%  Maximum missing data per sample – 5% |
| Validation | Ad hoc Perl script | |  |
| Phylogeny | dnapars, Consense | PHYLIP v3.69 | See text |
| Tree visualisation | FigTree v1.4.0 | |  |
| Haplogroup assignment | AMY-tree v1.2 | |  |
